# The Cardiac Neural Crest Cells in Heart Development and Congenital Heart Defects

**DOI:** 10.3390/jcdd8080089

**Published:** 2021-07-30

**Authors:** Shannon Erhardt, Mingjie Zheng, Xiaolei Zhao, Tram P. Le, Tina O. Findley, Jun Wang

**Affiliations:** 1 Department of Pediatrics, McGovern Medical School at UTHealth, The University of Texas Health Science Center at Houston, Houston, TX 77030, USA; shannon.erhardt@uth.tmc.edu (S.E.); mingjie.zheng@uth.tmc.edu (M.Z.); xiaolei.zhao@uth.tmc.edu (X.Z.); tram.p.le@uth.tmc.edu (T.P.L.); tina.o.findley@uth.tmc.edu (T.O.F.); 2 The University of Texas MD Anderson Cancer Center UTHealth Graduate School of Biomedical Sciences, The University of Texas Health Science Center at Houston, Houston, TX 77030, USA

**Keywords:** neural crest cells (NCCs), cardiac neural crest, heart development, outflow tract (OFT), congenital heart defects (CHDs)

## Abstract

The neural crest (NC) is a multipotent and temporarily migratory cell population stemming from the dorsal neural tube during vertebrate embryogenesis. Cardiac neural crest cells (NCCs), a specified subpopulation of the NC, are vital for normal cardiovascular development, as they significantly contribute to the pharyngeal arch arteries, the developing cardiac outflow tract (OFT), cardiac valves, and interventricular septum. Various signaling pathways are shown to orchestrate the proper migration, compaction, and differentiation of cardiac NCCs during cardiovascular development. Any loss or dysregulation of signaling pathways in cardiac NCCs can lead to abnormal cardiovascular development during embryogenesis, resulting in abnormalities categorized as congenital heart defects (CHDs). This review focuses on the contributions of cardiac NCCs to cardiovascular formation, discusses cardiac defects caused by a disruption of various regulatory factors, and summarizes the role of multiple signaling pathways during embryonic development. A better understanding of the cardiac NC and its vast regulatory network will provide a deeper insight into the mechanisms of the associated abnormalities, leading to potential therapeutic advancements.

## 1. Introduction

Neural crest cells (NCCs) are a multipotent, and highly migratory, transient vertebrate cell population originating in the dorsal region of the neural tube. During embryogenesis, NCCs, upon neural plate folding, arise from either side of the neural plate at a region called the neural plate border, situated between the neuroectoderm and non-neuroectoderm [1,2]. During and after the neural plate closes, NCCs undergo epithelial-to-mesenchymal transition (EMT) in which they obtain their migratory potential and disperse from the neural tube, relocating to specific locations throughout the embryo, to differentiate into a wide variety of cell types, such as osteoblasts and smooth muscle cells [2,3,4]. Although NCCs arise sequentially during embryo development, they are specified into four main subpopulations based on their anteroposterior axis position, differential abilities, and corresponding terminal locations [5]: cranial neural crest (NC), contributing to the majority of bone and cartilage formation of the head [6]; vagal NC, aiding in the formation of the thymus, lung, enteric nervous system and cardiovascular system [7]; trunk NC, contributing to the peripheral nervous and endocrine systems [8]; sacral NC, aiding in the development of neurons and glia of the enteric nervous system [9]. However, studies on these subpopulations have indicated that the vagal NC consists of a smaller specified group of cells deemed the cardiac NC, known to significantly contribute to cardiovascular development, along with aiding in the development of the thymus, thyroid glands, and cardiac ganglia [7,10,11,12,13].

NC ablation studies have demonstrated the importance of NCCs for proper heart formation. Deficiencies in these cells result in a variety of cardiac malformations during embryonic development categorized as congenital heart defects (CHDs) [14]. CHDs are the most common birth defect and the leading cause of birth defect-related deaths [15,16,17], with a variety of phenotypes ranging from mild forms, accompanied by minimal cardiac complications, to severe and life-threatening forms, resulting in extreme cardiac impediments and death. The most common defects, seen in approximately 30% of human cases, are atrial and ventricular septal defects (ASD/VSD), caused by a hole along the interatrial septum or interventricular septum, respectively [18]. Other common CHDs include malformations of the cardiac outflow tract (OFT), which normally gives rise to the proper vessel development and septation of the semilunar valves (leading to the aorta and pulmonary valves). OFT defects result in common CHD phenotypes such as transposition of the great arteries (TGA), aortic or pulmonary artery stenosis, and patent ductus arteriosus (PDA), as well as rarer CHD phenotypes, such as persistent truncus arteriosus (PTA), characterized by the absence of aortopulmonary septation, overriding aorta, in which the aorta is centralized over the VSD and open to both ventricles, and double outlet right ventricle (DORV), where the aorta is connected to the right, instead of left, ventricle [19,20,21]. Multiple congenital human syndromes, such as CHARGE, Treacher Collins, and DiGeorge, are associated with a variety of CHD phenotypes. However, how failure in proper NC development causes CHDs is still under investigation.

Although substantial progress has been made in uncovering the origin of congenital cardiac diseases, the underlying mechanisms controlling NCCs in heart development remain to be further elucidated. This review will focus on the recent discoveries in the contribution of cardiac NCCs for cardiac development, and how genetic alterations of various signaling pathways and transcription factors produce cardiac NC-related CHDs. A more comprehensive understanding of the roles NCs have in the formation of various cardiac structures, along with details regarding related mechanisms, will help illuminate the underlying causes of CHDs, providing novel advancements for genetic screenings and genetic-based therapies.

## 2. Contributions of the Cardiac Neural Crest to Cardiac Formation

The highly multipotent and temporarily migratory NC is a vertebrate-specific cell population divided into four main subpopulations, based on their migratory path, terminal location, and differential abilities: cranial, vagal, trunk, and sacral. During gastrulation, NCCs are induced from the dorsal region of the neural plate border, orchestrated by a variety of regulatory pathways such as Bmp, Wnt, Notch, and more recently, Hippo [22,23,24]. NC progenitors obtain their migratory potential by undergoing EMT [25]. Previous studies have indicated vital contributions of various signaling pathways such as Bmp, Wnt, and retinoic acid in regulating NC EMT and migration for proper cardiovascular development, but further investigation into cross-talk between signaling networks is needed [26].

In mammals, cardiac NCCs, a specified portion of the vagal NC and directly located below the cranial NC, initially migrate from the neural tube to pharyngeal arches three, four, and six, remodeling to form the great arteries, including the aortic arch, common carotid, and ductus arteriosus [27,28,29]. While a portion of cardiac NCCs remains within the arch arteries to assist in great artery repatterning and smooth muscle formation [28,30], the remaining cardiac NCCs maintain their migratory potential and travel to the cardiac OFT, a region formed during heart tube elongation and looping [31]. Shown in chicks and quail-chick chimeras, at the OFT, cardiac NCCs assist in forming the aorticopulmonary septum, creating a separation of the aorta and pulmonary trunks [31,32]. Cardiac NCCs also contribute to cardiac OFT cushion remodeling to form the semilunar valves (aorta and pulmonary valves), along with a portion of the interventricular septum in mice and chicks [32,33]. It has been suggested that cardiac NCCs can contribute to the interstitial cells of the OFT valves in zebrafish [34], however, further studies are needed to confirm such potential contributions, along with identifying their role in heart development and function.

The contribution of cardiac NCCs has been studied in a variety of model systems by the use of various techniques including specific NC ablation and lineage-tracing. Much of what is known about NC contributions to cardiac development originated from chick ablation and quail-chick chimera studies [35,36,37]. It was found that the combinational ablation of NC progenitors of pharyngeal arches one through six in chicks, resulted in a variety of CHD phenotypes such as VSD, PTA, and aortic arch abnormalities [35,36]. However, to determine how the cardiac NC contributes to heart development in mammals, various mouse models were constructed and showed similar migration and contribution patterns to what was seen in chick models [38]. Although both the bird and mouse (amniotes) have separated systemic and pulmonary circulatory systems, along with a four-chamber heart (two ventricles, two atria), mice showed varying contributions regarding cardiac NCC timing for OFT development [39]. In addition to ablation models, lineage tracing, which identifies and tracks cell populations in vivo, provides an advanced way to uncover NC-derived cardiovascular contributions. Ablation and lineage tracing experiments have shown that cardiac NCCs in amniotes originate between the optic vessel and third somite, and migrate to pharyngeal arches three, four and six, to contribute to smooth muscle development, septation of the OFT, and patterning of the septa, valves, and aortic arch arteries [7,40,41]. Recently, Tang and colleagues, using retroviral labeling in chicks and *Wnt1-Cre* reporter lines in mice, found that a portion of NCCs also contributes to ventricle trabecular myocardium, however, further and more in-depth lineage mapping of cardiac NCCs is needed to confirm these conclusions [42].

Although CHDs are mainly categorized by structural malformations, cardiac conduction system deficiencies have been linked to various cardiac defects such as VSD, along with CHD conditions such as tetralogy of fallot (TOF) [43]. It has been shown that cardiac NCCs give rise to portions of the cardiac conduction system. For example, Nakamura and colleagues found that NCCs marked with enhanced green fluorescent protein (EGFP) were colocalized with the conduction system-specific marker neurofilament 160 (NF160) in areas such as the His bundle and bundle branches of embryonic day (E) 17.5 mice [44], suggesting a role for NCCs in conduction system maturation. Such hypotheses are supported by Gurjarpadhye and colleagues who, by using an avian NC ablation model, showed the inhibition of conduction system function [45]. In contradiction to such conclusions, Miquerol and colleagues excluded the contribution of cardiac NCCs in mice to the ventricular conduction system (VCS), composed of the atrioventricular node and right and left bundle branches, due to finding no overlap of β-gal (beta-galactosidase) immunofluorescent or X-gal stained NCCs, with GFP-positive VCS cells [46]. Although there are conflicting conclusions regarding cardiac NCC contributions to the cardiac conduction system, which may stem from varying lineage tracing methods and model systems, such results indicate that the relationship between cardiac NCCs and the cardiac conduction system warrants further investigation.

In contrast to amniotes, the zebrafish heart has a single atrium and ventricle, along with a single-loop circulatory system that does not require OFT septation. Using fluorescein-based fate mapping and transgenic constructs, it was found that NCCs in the zebrafish originate from a higher axial level [47]. Recently, Cavanaugh and colleagues found that in zebrafish, NCCs that migrated to pharyngeal arches one and two contributed to heart tube myocardium, while NCCs from pharyngeal arch six contribute to the ventral aorta and bulbus arteriosus [48]. It was recently uncovered, by the use of lineage-tracing, that a portion of cardiac NCCs in zebrafish contribute to cardiomyocytes, however, the contribution of NCCs for cardiac development in zebrafish is still under investigation [43,48,49]. Similar to zebrafish, *Xenopus laevis* contain a single ventricle in a three-chambered heart, along with the incomplete septation of the OFT, which is not contributed by cardiac NCCs [50,51]. Yet, similarly to amniotes, cardiac NCCs in *Xenopus* contribute to the aortic arch arteries and the aortic sac [51,52].

Studies indicate the pivotal role of NCCs in cardiovascular formation. However, the varying contributions of NCCs for cardiac development, between multiple model systems, also suggest the need for further investigation into model-specific NC contributions and bring into question the benefits and disadvantages of using such model systems to study cardiac NC-derived cardiovascular defects and human cardiac diseases.

## 3. NC Associated Cardiac Congenital Abnormalities in Humans

Cardiac NC deficiency phenotypes are consistent with the CHDs present in a variety of human diseases. In recent years, the prevalence of CHDs has increased due to improved pediatric screening and increased survival [18]. However, further investigation is needed to determine the pathogenic and/or novel genetic deficiencies contributing to these cardiac defects, to improve diagnostic screening and treatment. Although many CHDs are considered nonsyndromic, multiple genetic syndromes, including DiGeorge and CHARGE, involve NC deficiencies with known associated cardiac phenotypes. Not only do NC deficiencies produce heart defects, they are also known to contribute to various craniofacial defects common among human cardio-craniofacial syndromes, such as the most common multiple anomaly syndromes DiGeorge, Noonan, and Velo-cardio-facial [53,54]. However, more studies are needed to determine how cardiac NC contributions are similar and/or different between multiple syndromes, and what NC regulatory pathways and factors are altered in such diseases. 

### 3.1. DiGeorge Syndrome

**Figure 1:**
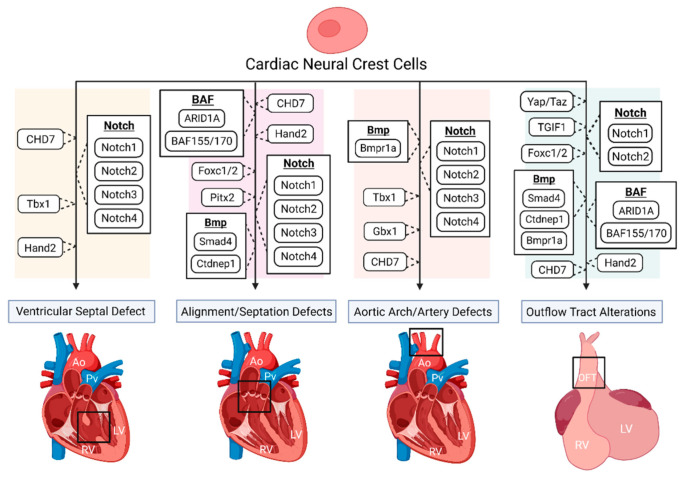
The disruption of various genes and signaling pathways, indicated to be important for proper cardiac neural crest (NC) contribution, results in numerous congenital heart defect (CHD) phenotypes. Such phenotypes include ventricular septal defect (VSD), various vascular defects including misalignment of the aorta and pulmonary trunks and aortic artery abnormalities, and outflow tract (OFT) alterations, such as OFT constriction and improper septation. Loss of *Tbx1* in mice has been shown to alter the environment of migrating neural crest cells (NCCs), producing great vessel abnormalities and aortic arch interruption, while also presenting with VSD in patients. *CHD7* mutations within NCCs can produce septum defects such as VSD, and vascular alterations such as double outlet right ventricle (DORV) and interrupted aortic arch, along with inhibiting NC contributions to the OFT. In NCCs, disrupted *Notch2* results in a narrowed OFT and OFT arteries, while mutations of all Notch-target genes (*Notch1*, *Notch2*, *Notch3*, *Notch4*) have been shown to result in VSD, along with vascular abnormalities such as pulmonic stenosis and aortic arch patterning defects. The deletion of a known bone morphogenetic protein (Bmp) inhibitor, *Ctdnep1*, produced premature and asymmetric OFT septation, pulmonary closure, premature NCC compaction, and embryonic death, while the loss of the Bmp receptor *Bmpr1a*, resulted in shortened OFTs leading to persistent truncus arteriosus (PTA), along with occasional aortic arch abnormalities. It was also found that the deletion of *Smad4*, located downstream of the Bmp network, also resulted in shortened OFTs, along with some hearts presenting with no OFT septation, resulting in PTA. The ablation of *Yap* and *Taz*, key components of the Hippo signaling pathway, in NCCs have been shown to disrupt smooth muscle expression and differentiation of the OFT. Deletions of *BAF155* and *BAF170* in NCCs have resulted in impaired NCC contribution for OFT development, while a loss of *ARID1A*, a BAF (Brg1/Brm-associated factors) subunit, has shown improper NC contribution for OFT development with producing conotruncal septation defects. The disruption of *Pitx2* and Pitx2 upstream regulators suggests a negative impact on cardiac NC abilities for proper vasculature development. *Hand2* deletions in NCCs present with a large variety of altered cardiac morphology, such as impaired OFT development, DORV, and VSD. Lack of OFT septation is also seen with the deletion of *TGIF1* in NCCs. Deletion of *Foxc1/2* in mice presents with lacking or shortened OFTs, along with improper OFT septation and PTA. Ao, aorta; Pv, pulmonary vessel; LV, left ventricle; RV, right ventricle; OFT, outflow tract. Created with Biorender.com (accessed on 28 July 2021).

DiGeorge syndrome, also known as 22q11.2 deletion, has a well-defined phenotype consisting of characteristic facial features, immunodeficiencies, CHDs, hypocalcemia, and developmental delays. Commonly associated CHDs include interrupted aortic arch, VSD, TOF, and PTA [55,56,57]. *Tbx1*, a region of Df1, the first targeted region homologous to human 22q11, is expressed in the pharyngeal region and is necessary for OFT and aortic arch development [58,59]. Although *Tbx1* has shown not to be expressed by NCCs [58,60], the loss of *Tbx1* has shown to be associated with VSD and TOF in patients [61]. Calmont and colleagues found that in mice, *Tbx1* drives downstream expression of genes such as *Gbx2*, to regulate cardiac NCC migration to the pharyngeal arches by a non-cell-autonomous effect, and that the combinational disruption of *Tbx1* and *Gbx2* results in abnormal pharyngeal arch development, along with aortic arch interruption (Figure 1), suggestively due to cardiac NC migration deficiencies [62]. However, further studies are needed to determine the specifics regarding the onset and longevity of such NC migration deficiencies, along with determining whether other various CHD phenotypes arise. It has been shown that a loss of *Tbx1* in mice, negatively impacts the development of the second heart field (SHF), partly due to a lack of cell proliferation, which can produce OFT hypoplasia and vascular trunk mislocalization [63,64]. SHF progenitors are a multipotent cardiac progenitor population important for heart tube looping, along with myocardium, smooth muscle, and endothelial cell formation. Given that SHF progenitors and cardiac NCCs closely interact and make major contributions to the development of the OFT, it is of great interest to determine the impact of such *Tbx1* depletions in the SHF on cardiac NCCs during heart development.

### 3.2. CHARGE Syndrome

CHARGE syndrome affects multiple organ systems and is an acronym for coloboma, heart defects, atresia choanae, growth and mental retardation, genital abnormalities, and ear abnormalities [65]. The most common CHD associated with CHARGE is TOF, detected in approximately 33% of human cases, followed by VSD and aortic arch abnormalities, suggesting that NC development is possibly affected during embryogenesis [66]. A study by Bajpai and colleagues showed that *CHD7*, the only mutated gene known to cause CHARGE syndrome, is essential for NC migration and the promotion of key transcriptional regulatory genes such as *Sox9*, *Twist1*, and *Slug*, both in vivo (*Xenopus*) and in vitro (human embryonic stem cells), while a deficiency of *CHD7* caused cardiac OFT defects in *Xenopus* embryos, resulting in vascular septation defects such as PTA (Figure 1) [67]. A recent study by Yan and colleagues found that the deletion of *CHD7* in NCCs of mice, by the use of the *Wnt1-Cre2* neural crest-specific driver, not only results in severe conotruncal defects (VSD and DORV), interrupted aortic arch, and perinatal lethality, but inhibits the OFT invasion of cardiac NCCs (Figure 1) [68]. The authors suggest that such cardiac defects are due to the establishment that CHD7 directly regulates key NC regulatory genes such as *Foxc2* and *Hand2*, through ATP-dependent and -independent functions, clarifying the molecular etiology of CHD7-related cardiac defects [68].

### 3.3. Treacher Collins Syndrome

Cardiac malformations can occur in patients with Treacher Collins syndrome. Treacher Collins is caused by a mutation within the *TCOF1* gene. TCOF1 is involved in mRNA formation in NCCs during embryogenesis, largely associated with NC depletions in pharyngeal arches 1 and 2 [69]. Treacher Collins is normally associated with craniofacial abnormalities, but the depletion of NCCs has also been indicated in producing human CHD phenotypes such as VSD, ASD, PDA [70]. Studies have found that haploinsufficiency of *TCOF1* in mice results in a reduced number of migrating NCCs, leading to severe craniofacial defects [69]. More recently, Sanchez and colleagues found that the knockdown of one of the only three genes known to be involved in Treacher Collins, *POLR1B* (RNA polymerase1 subunit B), results in notable cardiac edema, reduced NC migration, and embryonic death in zebrafish [71]. Serrano and colleagues, using human pluripotent stem cell (HPSC)-derived NCCs, found that disruption of *TCOF1*, by siRNA, confirmed previous conclusions that NC migration is impaired, but also found that NC proliferation was reduced [72]. Although many in vivo TCOF1 studies focus on cranial NC contributions, their findings, along with those from in vitro experiments, indicate a potential role of TCOF1 in cardiac NC function and cardiac formation, which requires further investigation.

## 4. Multiple Regulatory Pathways in NC-Derived CHDs

NC deficiency studies have provided vital information regarding NC contributions to cardiovascular development. Simultaneously, these studies indicate key genetic alterations within cardiac NCCs that contribute to CHDs. Deficiencies of various signaling pathways in NCCs, consistently result in CHD phenotypes such as improper OFT septation (PTA, DORV), VSD, and abnormal aortic arch patterning (interrupted aortic arch) [73,74]. Such genetic deficiency models provide vital evidence for the regulatory contributions of a variety of pathways for NC-derived heart development and CHD formation.

### 4.1. Notch Signaling Pathway

Vital for proper NC development, the Notch signaling pathway is highly conserved and required for multiple developmental processes such as cellular proliferation and specification [75]. It has been shown that mutations of Notch-target genes, such as *Notch1* and *Notch2*, along with Notch ligands such as *Jagged1*, in mice, cause a variety of heart defects such as VSD and malformations of the cardiac OFT and great vessels [76,77,78]. A study by Varadkar and colleagues found that Notch2is required for proper NC-derived aortic and pulmonary smooth muscle formation, and disrupted *Notch2* in post-migratory NCCs, using the *Pax3-Cre* driver, results in a narrowed OFT and OFT arteries (aorta and pulmonary) in E17.5 mice embryos (Figure 1), indicating a cell-autonomous role for Notch2 in NCCs [78]. In a supporting study, the inactivation of all four Notch receptors (*Notch1*, *Notch2*, *Notch3*, *Notch4*), by the dominant-negative version of MAML proteins (DNMAML), suppressed cardiac NCC differentiation into smooth muscle cells at E17.5 and E18.5 in mice, resulting in a variety of cardiac morphology inadequacies including VSD, pulmonary artery stenosis, and aortic arch-patterned defects (Figure 1) [77]. Although a conclusion from both of these studies was that Notch signaling is not required for cardiac NC migration [77,78], they pose varying conclusions to how Notch deficiencies disrupt smooth muscle differentiation in the developing heart, warranting further investigating of how Notch manipulation contributes to cardiac NC-derived heart development.

### 4.2. Bmp Signaling

One highly studied contributor to NC function is the signaling of bone morphogenetic proteins (Bmps). Bmps are proteins belonging to the transforming growth factor-beta (TGF-β) family, known to contribute to bone and cartilage formation. Bmps are crucial for neural plate border induction, along with influencing NC migration, survival, and differentiation [79,80,81]. Recent evidence indicated that Bmps also play an important regulatory role in heart function and development [82,83,84]. In addition to studies in the SHF, research also focused on the role of Bmps in NC-derived OFT septation, as improper OFT remodeling results in vessel defects that are consistent with the phenotypes seen in TOF patients. It is known that cardiac NCCs contribute to the formation of the semilunar valves via OFT septation and communicate with various cell populations. A recent study by Darrigrand and colleagues, in which *Dullard* (*Ctdnep1*), a nuclear phosphate known to inhibit Bmps, was deleted in NCCs of mice, resulted in premature and asymmetric OFT septation, pulmonary closure (Figure 1), and embryonic death, due to an increase in Smad1/5/8 activity [85]. Additionally, the removal of *Ctdnep1* in mice significantly decreased the expression of *Twist1*, *Snai1*, *Rac1*, *Mmp14*, and *Chd2*, known mesenchymal markers, resulting in premature NCC compaction [85]. Stottmann and colleagues found that the ablation of the Bmp receptor 1A (*Bmpr1a*) in NCCs, by *Wnt1-Cre* in mice, resulted in shortened OFTs with septation defects (PTA), embryonic lethality due to acute heart failure, and occasional hypoplastic aortic arch arteries, indicating that Bmp signaling is required for proper OFT development (Figure 1) [86]. The authors suggest that NCCs need Bmpr1a for OFT colonization and that such phenotypes are caused by NCCs lacking downstream factors of Bmpr1a needed for the continued development of the OFT [86]. Studies have also shown that the ablation of *Bmp4* in the myocardium of mice, results in early embryonic lethality, along with severe OFT septation, interventricular septum alterations (VSD), and semilunar valve defects (PTA), suggesting that Bmps, may signal to cardiac NCCs, for proper OFT development and division [87,88]. Downstream of Bmp signaling, Smad transcription factors, also a component of the TGF-β network, have been shown to impact NC-derived cardiac development. It has been shown that *Smad4* deficiencies, by *Wnt1-Cre* in mice, resulted in a shortened OFT, and no OFT septation, resulting in a PTA phenotype (Figure 1), likely due to a reduced number of cardiac NCCs and a reduced expression of genes such as *Id1*, *Id3*, *Msx1*, and *Msx2* [89].

### 4.3. Hippo-Yap Signaling Pathway

A more recently discovered pathway with a potential role in regulating proper NC-derived cardiac formation is the Hippo signaling pathway. The Hippo signaling pathway is essential for the regulation of organ size and development [90]. Wang and colleagues demonstrated that the specific ablation of the Hippo pathway co-effectors, *Yap* and *Taz*, using *Wnt1-Cre* and *Wnt1-Cre2SOR* in the NCCs of mouse embryos, resulted in embryonic lethality with severe craniofacial vascular defects, and blood vessel defects in the branchial arches of E10.5 mouse embryos; however, the underlying mechanism is still unknown [91]. Similarly, Manderfield and colleagues found that the deletion of *Yap* and *Taz* in NCCs impaired NCC-derived smooth muscle cell differentiation of E10.5 mouse embryos (Figure 1), yet this complete deletion did not affect normal cardiac NC migration to the branchial arches and peripheral ganglia [92,93]. Yap/Taz-deficient NC studies in mice have posed a challenge due to *Yap/Taz* complete deletion resulting in early embryonic lethality, but indicate the need for a better understand of Hippo signaling in controlling proper NCC contributions for cardiac formation [91,92,93]. In addition, recent studies indicated a positive role of Yap in promoting NC migration in avian embryos [94,95], suggesting its varying role of dependency between different model systems, highlighting the need of further investigations.

### 4.4. BAF Signaling Complex

BAF (Brg1/Brm-associated factors), an ATP-dependent chromatin remodeling complex, regulates multiple NC processes vital for cardiac development. It was previously found that in mice, the conditional loss of the BAF subunit *ARID1A* (AT-rich interactive domain-containing protein 1A), a known tumor suppressor, led to altered cardiac NC migration, improper NC colonization to the OFT, and impaired conotruncal septation (Figure 1), potentially due to ARID1A-contain BAF complexes being the predominant form of the SWI/SNF complex in cardiac NCCs [96]. Bi-Lin and colleagues found that the deletion of the BAF core components, *BAF155* and *BAF170*, in mice, resulted in impaired contributions of NCCs to the developing OFT, a decrease in NCC differentiation in smooth muscle cells, and an increase in apoptosis in the pharyngeal arch arteries, indicating a novel role for this complex in NC-derived CHDs (Figure 1), possibly through the interaction with other signaling pathways such as Hippo and Notch [97]. Additional genetic studies focusing on the impact of various BAF components, along with crosstalk between pathways, on cardiac NCCs and heart development will potentially lead to clarity in regard to CHD phenotype-based genetic alterations.

### 4.5. Transcription Factors Indicated in NC-Derived Heart Development and Associated CHDs

In an effort to better understand the vast regulatory network impacting NC-derived heart development, several transcription factors have been identified as vital, and their removal has resulted in various CHD phenotypes. For example, Ganhdi and colleagues determined that TGFB-induced factor homeobox 1 (TGIF1), previously unattributed with cardiac NCCs, is vital for NC specification and correct OFT septation, due to its knockout by a CRISPR-Cas9 mediated strategy, reducing known NC markers (*Tfap2B*, *CXCR4*) in chicks, and producing a single OFT vessel that failed to septate in quail-chick chimeras (Figure 1) [98]. The authors suggest that TGIF1 comprises a transcriptional subcircuit, with transcription factors Ets1 and Sox8, that is vital for cardiac NCCs and proper heart development [98]. A previous study in mice showed that the deletion of *Pitx2*, known to be expressed in cardiac NCCs, results in CHD phenotypes such as PTA, transposition of the great arteries, and DORV, due to the establishment of Pitx2 being an important target of the Wnt signaling pathway in regard to cardiac development (Figure 1) [99]. Extending from this work, it was further indicated that *Dlv2* (*Dishevelled 2*), part of the Wnt pathway, is potentially required for Pitx2 in migrating NCCs of mice, due to the deletion of *Dlv2* resulting in a substantial reduction in *Pitx2* expression in migrating cardiac NCCs and severe cardiac defects (DORV, PTA), suggesting that Wnt/Dvl serves as a regulator of Pitx2 expression for cardiac NC-derived heart development [99,100]. It is known that both Hand1 and Hand2 (heart and neural crest derivatives expressed 1 and 2) are present in NCCs and NC-derived structures [101]. Hand2 was identified by Holler and colleagues as having a regulatory role in NC migration to the OFT and proper heart development, as the deletion of *Hand2*, specifically in NCCs by the use of the *Wnt1-Cre* driver in mice, resulted in DORV and VSD, along with negatively impacting cardiac NC migration to the OFT (Figure 1) and expression of genes associated with other pathways such as sonic hedgehog (*Gli3*) and Notch (*Hey1*, *Foxc1*) [102]. Similarly, to Hand1/2, Foxc1 and Foxc2 (Forkhead Box C1 and 2) are expressed in NCCs and NC-derived structures [101]. It was found that the complete ablation of both *Foxc1* and *Foxc2* of E9.0 mice, resulted in embryonic death accompanied by a complete lack of the OFT, while the mutants with the complete deletion of Foxc2 and a partial deletion of Foxc1 presented with hypoplastic OFTs, along with failed OFT septation and PTA, due to an increase in apoptosis of cardiac NCCs and a decreased contribution of NCCs to the OFT (Figure 1) [103].

Although many pathways and transcription factors have been linked to NC-derived CHDs, such as those mentioned in this review, research is currently being conducted to uncover various other factors needed for proper NC-derived cardiovascular formation. However, research is also needed to determine how the cross-talk of these various signals impacts proper cardiac NC contribution, to better understand the onset of CHDs.

## 5. Conclusions and Future Perspective

NCCs are known for their multipotent and migratory potentials, contributing to various cell types for organ and tissue development. It is well-known that the cardiac NC is highly regulated by a variety of pathways, and that insufficient NC contribution to the heart results in CHDs involving the OFT, great vessels, and cardiac septa.

The use of altered genetic models and cell labeling has provided vital information on how various signaling pathways regulate NC function and contribute to cardiac development. Although progress has been made in understanding the regulatory networks of NC-derived cardiovascular formation, NCC developmental processes are exceedingly complex. Further investigation is needed to determine the impact that known contributors have at different developmental stages, regarding NC specification, migration, and differentiation, and how genetic deficiencies of these pathways lead to CHDs. For example, the information gathered from cranial NC studies has led to further questions regarding how Hippo signaling regulates cardiac development through NCCs [91,92,93]. One area in need of further investigation is the Hippo signaling pathway’s role and impact on cardiac NC contributions to heart development. Although reports have indicated that NC migration to the pharyngeal arches was intact in *Yap/Taz*-deleted mouse embryos [91,92], further studies are needed to determine migration abilities from the arches to the developing OFT, along with differentiation capabilities throughout cardiac formation. Furthermore, investigation is warranted regarding the mechanisms resulting in early embryonic lethality, as seen in deficient Hippo and Bmp signaling pathways [87,91].

Recent studies showed that the dysregulation of various gene regulatory networks negatively impacts proper NCC function and is associated with human diseases such as CHARGE (*CHD7*), Treacher Collins (*TCOF1*, *POLR1B*), and DiGeorge (*Tbx1*) [62,66,69,71]. Although there are common cardiac congenital defects seen between these syndromes, mainly VSD and OFT malformations, each syndrome is the result of different regulatory deficiencies associated with cardiac NCCs. These findings indicate the critical role of NCCs in proper cardiac formation, while also highlighting the significance of studying NCC deficiencies in human diseases.

To enhance current therapies for patients with heart defects, various studies have begun investigating the potential role of NCCs for cardiac regeneration. Cardiac regeneration is currently at the forefront of cell-renewal research, with the effort to repair irreversibly damaged heart tissue. Cardiac NCCs are known to contribute to the cardiomyocytes of the trabecular myocardium in zebrafish, chicks, and mice [43,48,49]. Tang and colleagues have found a novel contribution of cardiac NCCs for zebrafish heart regeneration, by the reactivation of genes such as *sox10* and *tfap2a* after the surgical removal of a portion of the ventricle [43]. This novel regeneration capability of zebrafish cardiac NCCs poses the possibility for regeneration capability in amniotes; however, further examination is needed to determine whether this capability is indeed found in various species, along with the determination of the mechanisms regulating such renewal capabilities.

This review summarized the pivotal roles of NCCs in cardiac development during embryogenesis. NCCs warrant further investigation into how various genetic deficiencies result in their disrupted function in cardiac formation, to provid novel information regarding the formation of CHDs.

## Abbreviations

NCC, neural crest cell; EMT, epithelial-to-mesenchymal transition; NC, neural crest; CHDs, congenital heart defects; ASD, atrial septal defect; VSD, ventricular septal defect; OFT, outflow tract; TGA, transposition of the great arteries; PTA, persistent truncus arteriosus; DORV, double outlet right ventricle; TOF, tetralogy of fallot; VCS, ventricular conduction system; SHF, second heart field; PDA, patent ductus arteriosus.
